# The role of environmental feedback in a brain state switch from passive to active sensing

**DOI:** 10.1186/1471-2202-14-S1-P395

**Published:** 2013-07-08

**Authors:** Christopher L Buckley, Taro Toyoizumi

**Affiliations:** 1 Lab for Neural Computation and Adaptation, Riken Brain Science Institute, Tokyo, Japan

## 

Coherent behaviour emerges from mutual interaction between the brain, body and environment across multiple timescales and not from within the brain alone [1,2]. For example sensation is actively shaped by dynamical interaction of the brain and environment through motor actions such as sniffing, saccading, and touching. The onset of active sensing is often concomitant with qualitative changes in neural dynamics [3] and responses to sensory input [4,5]. Indeed, some neural responses are uniquely sensitive to the presence or absence of dynamical sensory feedback [6]. However, understanding how active sensing strategies impact on neural dynamics and sensory responses is an open challenge. Whisking behaviour in rodents has been the central model system for studying neural mechanisms of active sensing. During quiet wakefulness the membrane potential of neurons in the barrel cortex exhibit high power, low frequency fluctuations and nearby neurons become highly correlated [3]. As active whisking onsets, the brain state of the barrel cortex qualitatively changes: low frequency fluctuations are suppressed and nearby neurons decorrelate [3]. Interestingly both correlation and low frequency LFP are partially restored during periods of active touch, i.e., when the mouse palpate towards, and repeatedly contacts with an object [3]. The brain state transition is also concomitant with changes in responses of barrel cortex neurons to whisker stimulation. The sensitivity of neurons to whisker perturbation drops with the onset of whisking [5]. However, robust and repeatable whisker response are recovered for more naturalistic stimuli, i.e., active touch [5].

Here we propose a theory, and construct a model, of active whisking that explains the core phenomenology through the dynamical interaction between the brain and the environment. The three core assumptions underlying the theory are:

1. Strong low frequency membrane fluctuations, intra-neural correlations between nearby neurons, and sensory responses to brief whisker deflection associative with quiet attentive state arise as result of network dynamics that are close to a dynamical instability.

2. Reafferent input (sensory feedback related to self-action) during whisking behaviour provides negative feedback to sensory neurons that stabilize cortical dynamics reducing low frequency fluctuations, intra neural correlations, sensory responses to brief whisker deflection, while increasing the correlation between cortical activity and whisker position.

3. Interrupting the reafferent signal, via a whisker touch event, temporally destabilise the cortex and enhances ex-afferent input (external sensory input coding whisker contacts).

Our theory and model suggests that sensory feedback via reafference signals is *sufficient *to account for the changes in cortical dynamics that appear with the onset of active whisking and provides a novel mechanistic account for sensory processing during active whisking. We discuss how this feedback stabilization mechanism coexists and interacts with other internal mechanisms that modulate cortical dynamics within the brain [3,7]. We quantify how this mechanism of active touch enhances the signal-to noise ratio of the input to the cortex. Finally we discuss the wider implications of these results for experimental work attempting to characterise neural activity and responses in an open-loop (in the absence of sensory/motor feedback) condition.
